# First Genome Sequences of Porcine Parvovirus 5 Strains Identified in Polish Pigs

**DOI:** 10.1128/genomeA.00812-16

**Published:** 2016-09-01

**Authors:** Jinghui Fan, Jin Cui, Priscilla F. Gerber, Kinga Biernacka, Tomasz Stadejek, Tanja Opriessnig

**Affiliations:** a The Roslin Institute and The Royal (Dick) School of Veterinary Studies, University of Edinburgh, Midlothian, United Kingdom; b College of Veterinary Medicine, Agricultural University of Hebei, Baoding, China; c Department of Pathology and Veterinary Diagnostics, Faculty of Veterinary Medicine, Warsaw University of Life Sciences, Warsaw, Poland; d Department of Veterinary Diagnostic and Production Animal Medicine, College of Veterinary Medicine, Iowa State University, Ames, Iowa, USA

## Abstract

Porcine parvovirus type 5 (PPV5) has been recently identified. Here, we report the genome sequences of five PPV5 strains identified in serum samples from Polish pigs, which represent the first PPV5 sequences recovered from European pigs. The PPV5 strains isolated in Poland are most related to the Chinese strain HN01.

## GENOME ANNOUNCEMENT

Porcine parvovirus (PPV) is a member of the subfamily *Parvovirinae* in the family *Parvoviridae* (1). PPV is a small nonenveloped DNA virus with a single-stranded linear genome of approximately 4.0 to 6.3 kb in size (2). The longest recognized parvovirus in pigs is PPV1, which belongs to the genus *Protoparvovirus* and which is commonly associated with reproductive failures in breeding herds. During the past decade, several novel PPVs have been identified (3), which have been designated PPV2 through PPV6. Unlike PPV1, these new emerging parvoviruses in pigs belong to one of the two new genera, *Tetraparvovirus* (PPV2 and PPV3) and *Copiparvovirus* (PPV4, PPV5, and PPV6). PPV5, initially detected mainly in grow-finish pigs in the United States in 2013 (4, 5) and subsequently also identified in China in 2014 (6), is most closely related to PPV4, with an overall genome similarity of 64.1% to 67.3%. Unlike PPV4, which has an additional open reading frame 3 (ORF3), PPV5 like other PPV strains has two ORFs. Here, we report the first PPV5 genome sequences identified in European pigs.

In the present study, 247 serum samples, collected from pigs of different ages in six farms as part of porcine reproductive and respiratory syndrome virus (PRRSV) surveillance, were used to investigate PPV5 circulation in Poland. Nucleic acids were extracted from serum samples and tested with a PPV5 real-time PCR assay (4, 7). PPV5 DNA was detected in four of six farms and in 4% (10/247) of the samples. Five of the 10 PCR-positive samples from two of the PPV5-positive farms were selected for sequencing and were designated K17-1, K17-4, P12-1, P13-9, and P13-10. Four overlapping fragments were amplified using a set of primers described previously (4, 5). The PCR products were purified using the PureLink quick gel extraction and PCR purification combo kit (Thermo Fisher Scientific) and sequenced by the ABI3730XL platform at Edinburgh Genomics (Edinburgh, United Kingdom). The obtained sequences were compared with published sequences by BLAST and assembled by the Lasergene software.

The complete coding regions of five PPV5 strains were 4,895 or 4,901 nucleotides (nt) in length, containing two major ORFs. The ORF1 (1,797 or 1,803 nt) encoded the nonstructural 1 (NS1) protein, with 599 or 601 amino acids (aa), and the ORF2 (2,973 nt) encoded VP1 protein, with 991 aa. These regions of the identified PPV5 strains shared high similarities (99.2% to 99.7%) with reference strains. The phylogenetic tree of the nearly complete genomes showed that all PPV5 strains isolated in Poland were related to the Chinese strain HN01. However, two unique amino acid deletions in the NS1 protein, which also occurred in Chinese strain HN01 (GenBank accession no. AF661535, aa positions 22 and 222), were observed in the PPV5 K17-4 strain. The significance of these amino acid deletions on the pathogenicity of PPV may be worth further investigation. In conclusion, the sequences of these five Polish PPV5 strains will help to understand the epidemiology and evolution of PPV in Europe.

### Accession number(s).

The genome sequences of PPV5 strains K17-1 (accession no. KX352458), K17-4 (accession no. KX273436), P12-1 (accession no. KX352457), P13-9 (accession no. KX352455), and P13-10 (accession no. KX352456) have been deposited in GenBank.
